# Promising Antimycobacterial Activities of Flavonoids against *Mycobacterium* sp. Drug Targets: A Comprehensive Review

**DOI:** 10.3390/molecules27165335

**Published:** 2022-08-22

**Authors:** Ali A. Rabaan, Saad Alhumaid, Hawra Albayat, Mohammed Alsaeed, Fadwa S. Alofi, Mawaheb H. Al-Howaidi, Safaa A. Turkistani, Salah M. Alhajri, Hejji E. Alahmed, Abdulwahab B. Alzahrani, Mutaib M. Mashraqi, Sara Alwarthan, Mashael Alhajri, Fatimah S. Alshahrani, Souad A. Almuthree, Roua A. Alsubki, Abdulmonem A. Abuzaid, Mubarak Alfaresi, Mona A. Al Fares, Abbas Al Mutair

**Affiliations:** 1 Molecular Diagnostic Laboratory, Johns Hopkins Aramco Healthcare, Dhahran 31311, Saudi Arabia; 2 College of Medicine, Alfaisal University, Riyadh 11533, Saudi Arabia; 3 Department of Public Health and Nutrition, The University of Haripur, Haripur 22610, Pakistan; 4 Administration of Pharmaceutical Care, Al-Ahsa Health Cluster, Ministry of Health, Al-Ahsa 31982, Saudi Arabia; 5 Infectious Disease Department, King Saud Medical City, Riyadh 7790, Saudi Arabia; 6 Infectious Disease Division, Department of Medicine, Prince Sultan Military Medical City, Riyadh 11159, Saudi Arabia; 7 Department of Infectious Diseases, King Fahad Hospital, Madinah 42351, Saudi Arabia; 8 Clinical Microbiology Division, Medical Laboratory Department, Qatif Health Network, Qatif 31911, Saudi Arabia; 9 Fakeeh College for Medical Science, Jeddah 21134, Saudi Arabia; 10 Infectious and Zoonotic Diseases Division, Ministry of Environment, Water and Agriculture, Al-Ahsa 11116, Saudi Arabia; 11 Department of Laboratory and Blood Bank, King Fahad Hospital, Al Hofuf 36441, Saudi Arabia; 12 Molecular Diagnostic Laboratory, King Fahad Armed Forces Hospital, Jeddah 21159, Saudi Arabia; 13 Department of Clinical Laboratory Sciences, College of Applied Medical Sciences, Najran University, Najran 61441, Saudi Arabia; 14 Department of Internal Medicine, College of Medicine, Imam Abdulrahman Bin Faisal University, Dammam 34212, Saudi Arabia; 15 Department of Internal Medicine, College of Medicine, King Saud University, Riyadh 11362, Saudi Arabia; 16 Division of Infectious Diseases, Department of Internal Medicine, College of Medicine, King Saud University and King Saud University Medical City, Riyadh 11451, Saudi Arabia; 17 Department of Infectious Disease, King Abdullah Medical City, Makkah 43442, Saudi Arabia; 18 Department of Clinical Laboratory Sciences, College of Applied Medical Sciences, King Saud University, Riyadh 11362, Saudi Arabia; 19 Medical Microbiology Department, Security Forces Hospital Programme, Dammam 32314, Saudi Arabia; 20 Department of Pathology and Laboratory Medicine, Sheikh Khalifa General Hospital, Umm Al Quwain P.O. Box 499, United Arab Emirates; 21 Department of Pathology, College of Medicine, Mohammed Bin Rashid University of Medicine and Health Sciences, Dubai P.O. Box 505055, United Arab Emirates; 22 Department of Internal Medicine, King Abdulaziz University Hospital, Jeddah 21589, Saudi Arabia; 23 Research Center, Almoosa Specialist Hospital, Al-Ahsa 36342, Saudi Arabia; 24 College of Nursing, Princess Norah Bint Abdulrahman University, Riyadh 11564, Saudi Arabia; 25 School of Nursing, Wollongong University, Wollongong, NSW 2522, Australia; 26 Nursing Department, Prince Sultan Military College of Health Sciences, Dhahran 33048, Saudi Arabia

**Keywords:** *Mycobacterium tuberculosis*, flavonoids, drug discovery, anti-tubercular compounds

## Abstract

Tuberculosis (TB) caused by the bacterial pathogen *Mycobacterium tuberculosis* (*Mtb*) remains a threat to mankind, with over a billion of deaths in the last two centuries. Recent advancements in science have contributed to an understanding of *Mtb* pathogenesis and developed effective control tools, including effective drugs to control the global pandemic. However, the emergence of drug resistant *Mtb* strains has seriously affected the TB eradication program around the world. There is, therefore, an urgent need to develop new drugs for TB treatment, which has grown researchers’ interest in small molecule-based drug designing and development. The small molecules-based treatments hold significant potential to overcome drug resistance and even provide opportunities for multimodal therapy. In this context, various natural and synthetic flavonoids were reported for the effective treatment of TB. In this review, we have summarized the recent advancement in the understanding of *Mtb* pathogenesis and the importance of both natural and synthetic flavonoids against *Mtb* infection studied using in vitro and in silico methods. We have also included flavonoids that are able to inhibit the growth of non-tubercular mycobacterial organisms. Hence, understanding the therapeutic properties of flavonoids can be useful for the future treatment of TB.

## 1. Introduction

Tuberculosis (TB) is a bacterial infection caused by bacterium called *Mycobacterium tuberculosis* (*Mtb*), and has been the biggest cause of death in the world. However, the development of COVID-19 in 2020 has surpassed tuberculosis as the major infectious illnesses of mortality [1]. WHO global TB report According to the 2021, there has been a decline in the TB incidence, notification, and an increase in the TB death rate [2]. The WHO 2021 TB report has provided insight into the dominance of the COVID-19 pandemic in both the scientific community and the media, resulting in the neglect of other communicable infections, including TB infection [1,3]. In general, the spread of the pandemic has seriously affected the progress of TB infection eradication programs worldwide, which need to be back on track with much greater effort.

Even though TB is curable, still it comes under a lethal infection, due to the prevalence of the airborne pathogen *Mtb* in the environment. The genus Mycobacterium has more than 170 species [4] and, only three species, *Mtb, Mycobacterium leprae*, and *Mycobacterium ulcerans*, are the known as predominant human bacterial pathogens. Several non-tuberculous mycobacteria, such as *Mycobacterium avium*, *Mycobacterium marinum*, *Mycobacterium xenopi*, *Mycobacterium gordonae*, and *Mycobacterium kansasii*, are also reported to cause disease in an individual having low immunity [5,6]. TB infection has been classified into two groups according to the mode of infection: pulmonary tuberculosis and extrapulmonary tuberculosis. Pulmonary TB infection occurs when the bacterial infection occurs in the lungs. It accounts for around 80% of total cases of TB infection. Extrapulmonary TB infection occurs when the pathogen infects other tissues of the body such as the colon, meninges, lymph nodes, bones, joints, kidneys, and skin [7]. It is seen that *Mycobacterium* sp. mainly infects those people who have weaker immune systems due to certain medical conditions such as HIV/AIDS, cancer, diabetes, malnutrition, liver sclerosis, organ transplantation, and also due to certain lifestyle disorders such as excessive alcohol and tobacco consumption, various fungal infections, and air pollution, etc., [8,9].

Currently, antibiotics prescribed for the treatment of TB patients include a combination of drugs including first-line drug and second-line anti-TB drug for a period of 6-month. The first-line group drugs rifampicin (RIF), isoniazid (INH), pyrazinamide (PZA), and ethambutol (EMB) are administered for 2 months, followed by a combination of RIF and INH for 4–6 months as core treatment regime for the active TB patients [10]. However, in the last few decades, TB treatment has been more challenging due to the emergence of drug-resistant strains of *Mtb*. Long-term administration of antibiotics, inadequate drug supply, and improper regimen selection develop certain mycobacterial strains resistant to drugs. When the mycobacterium genome gets mutated due to various factors, certain enzymes present in the bacteria become activated, causing structural changes in the drugs, which affect the inhibiting property of the given drug, resulting in drug-resistant tuberculosis, such as multidrug-resistant (MDR: resistant at least to INH and RIF antibiotics) and extensive drug-resistant tuberculosis [MDR resistant to a fluoroquinolone (FQ) and a second-line injectable drugs, kanamycin (KM), amikacin (AM), capreomycin (CM)] [11,12]. Although multiple anti-TB medications are produced using various drug discovery approaches, the target-based approach to drug discovery has played a vital role in identifying promising anti-TB drugs, particularly from natural compounds [13,14]. Anti-TB medicines found from natural sources are thought to be a better treatment method for dealing with drug resistance. Secondary metabolites derived from medicinal plants are also excellent sources from ancient times. Natural products such as alkaloids, coumarins, flavonoids, polyphenols, terpenoids, etc., have antimicrobial properties, that could be useful in the treatment of tuberculosis [15].

Flavonoids are one of the most extensively studied natural products derived from plants and fungi, and reported for numerous medicinal properties such as antimicrobial, antioxidant, anti-inflammatory, anti-cancer, and antiviral properties [15,16]. These natural compounds are also known to prevent infection by suppressing the growth of the pathogenic microorganism, including drug-resistant strains of *Mtb*, and strengthen the host immunity system [16]. Flavonoids belong to the class of phenolic compounds having a standard structure containing two phenyl rings attached to a heterocyclic ring [17]. Nevadensine, naringenin, isoflavanquinone, epicatechin, isoharmnetin, kaempferol, luteolin, myricetin, and quercetin are some of the examples of flavonoids with anti-tuberculosis activity. According to research, intravenous usage of rutin can treat pulmonary tuberculosis [18]. Anti-tuberculosis activity of synthetic flavanones has also been reported against *Mtb*. Based on the preceding considerations, this review emphasizes the importance of flavonoids as anti-tubercular compounds, as well as their bioactivity on several druggable targets of *Mtb* and other pathogenic *Mycobacterium* sp. Thus, to collect the data for the review, major databases, including PubMed and Google scholar, were searched with the key words such as ‘flavonoids’, ‘Anti-tuberculosis’, ‘tuberculosis’, and ‘anti-mycobacterial’ or ‘anti-bacterial’. The initial search was done on 20 June 2022 to find papers published between 2001 and 2022, followed by another search on 30 June 2022 to update and add the latest data to the review. After obtaining all the data from various keyword search, further manual refinement was carried out to extract most relevant papers. Herein, from >2000 search hits, more than 500 review papers were removed while the rest of search hits were divided into two groups, i.e., in vitro and in silico research papers. From these, papers related to non-mycobacterial disease were further removed and only those papers related to flavonoids as antimycobacterial or antitubercular agents, or flavonoids reported with activity against various mycobacterial proteins were selected for the final analysis. This results in collection of more than 100 flavonoids with antitubercular activity. Along with these, flavonoids against various non-tubercular mycobacterial disease were also considered to get better understanding on the activity of flavonoids. However, we have only added and discussed only flavonoids with relevant citation while the rest of the flavonoids with missing information were excluded from the manuscript. Additionally, articles with general data about *Mtb* and flavonoids targeting various antitubercular targets along with their mechanism were also discussed.

## 2. Biology of Tuberculosis Infection

Tuberculosis is caused by *Mycobacterium tuberculosis (Mtb)*, a Gram-negative, rod-shaped bacteria belonging to the family Mycobacteriaceae. Even though *Mtb* can infect other mammalian species, humans are their significant reservoir [19,20]. Tuberculosis infections arise when the bacterial growth spread into the air by infected patients coughing, sneezing, or close contact; these aerosol droplets enter the host’s alveoli via inhalation. This leads to the activation of the host’s innate immune system, resulting in *Mtb* phagocytosis by alveolar macrophages [21]. If the *Mtb* gets beyond the first line of defense, they start multiplying inside the macrophages and spread to the surrounding cells, including epithelial and endothelial cells, and reach their exponential growth phase in a few weeks [22]. Additionally, *Mtb* can also spread to other organs via the circulation or lymphatic system during the initial stage of illness [23].

**Figure 1:**
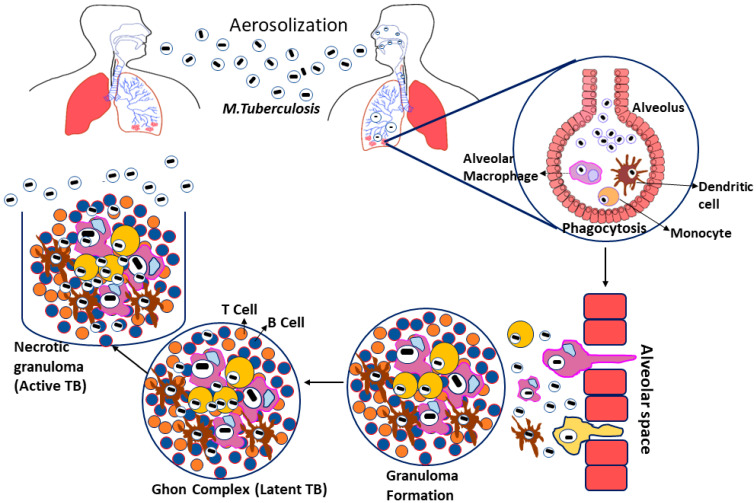
Pathogenesis of *Mycobacterium tuberculosis*.

Furthermore, infected macrophages in the lungs produce chemokines that attract inactivated monocytes, lymphocytes, and neutrophils, which do not effectively eradicate the *Mtb* infection [24]. When the adaptive immune response is initiated, the migrating neutrophils, lymphocytes, and monocytes aggregate to create a formation known as granuloma [25]. The granuloma is coated by a fibrotic component and becomes a calcified structure to protect the pathogens from the host immune response. They are considered as the primary lesion that develops in TB patients, also known as the Ghon complex [26]. The ghon complex is thought to be *Mtb*’s refuge during latent infection because the bacilli enter dormancy and remain metabolically inert for an extended length of time. So, given the right conditions, the *Mtb* infection may begin to proliferate within the complex leading to the activation of disease [27] (Figure 1).

### Mycobacterium tuberculosis-Drug Resistance Mechanism

The drug resistance condition in *Mtb* arises due to two major factors: extrinsic factors and intrinsic factors. Extrinsic factors are based on the total TB population and the quality of the TB prevention and control services in society, but intrinsic factors are associated with a genetic mutation in the genes coding for drug target or drug activation enzymes [28,29]. The genetic mutation mainly arises due to single nucleotide polymorphisms (SNP) or through the insertion or deletion of nucleotides [30,31]. Mutation in catalase-peroxidase gene (katG), inhA gene, or alkyl hydroperoxide reductase gene are responsible for inducing isoniazid (INH)-resistant tuberculosis [32]. Other mechanisms that contribute to drug resistance development include drug selection pressure, efflux pump mechanism, and epistasis [33]. Aside from target-based mutation, various alternative mechanisms of medication resistance have been documented. Some of these include structural drug modification by *Mtb* enzymes, drug inhibition to the target due to low permeability of the bacterial cell wall, drug removal due to activation of *Mtb* efflux pump, pathogen gene expression modulation to reduce growth, and shutting down the metabolic pathway to adapt the effect or presence of the drug [34].

## 3. Promising Therapeutic Strategies for the Treatment of Tuberculosis Infection

Although several antibiotic treatments play an important role in preventing the establishment of drug resistance tuberculosis, the continued use of drugs for an extended treatment period, combined with medication toxicity, increases the risks of the development of drug-resistant strains. In addition, problems such as a weakened immune system, HIV infection, or any other chronic disease lowered the likelihood of recovery. Given these facts, the necessity for a novel or improved therapeutic strategies should be seriously considered [35]. Natural products and their derivatives have shown promise in the treatment of a variety of infectious diseases, including tuberculosis. Plant-based medications can be generated efficiently, cost-effectively, and made readily available to the pharmaceutical market. Aside from that, the usage of natural compounds, such as flavonoids, can alleviate concerns about drug toxicity and drug resistance [36,37]. The most efficient therapeutic methods for tuberculosis therapy are the identification of several therapeutical targets to suppress the growth of the pathogen using anti-TB drugs [38]. Some of the potential treatment techniques explored to date include the inhibition of cell wall production, mycolic acid synthesis, arabinogalactan synthesis, RNA synthesis, DNA synthesis, and protein synthesis. Inhibition of cell wall synthesis mainly includes the inhibition of mycolic acid and arabinogalactan synthesis, as these two are the important components of the cell wall of *Mtb* [38,39]. Inhibiting the synthesis of these components may cause change in the cell wall permeability to drug transport and suppress pathogen development [39]. Moreover, host direct therapy (HDT) is another new approach for treating tuberculosis in which the host responses are controlled by utilizing small molecules in the presence or absence of adjunct antibiotics for better tuberculosis treatment. The HDT drugs directly influence the cell activities, increasing the possibility of treatment resistance to TB. Some potential HDT medications target includes granuloma structure, which allows drug penetration through the cell wall, autophagy induction, and improve intracellular bacterial death, while targeting anti-inflammatory cells to suppress inflammation and cellular breakdown. As a result of these considerations, host-directed therapeutic approaches have gained significant research interest as an addition to anti-TB drugs [35].

### 3.1. Flavonoids

Flavonoids are the largest phenolic phytochemical group. They are the secondary metabolites obtained from plants and a found in the non-glycosylated or glycosidic form. They are the most essential component in various nutraceutical, pharmaceutical, medicinal, and cosmetic industries [40]. Over 9000 structures of flavonoids have been reported showing antioxidant, anti-inflammatory, anti-bacterial, and anti-cancerous properties [41]. Flavonoids are also found in plant-based food and beverages such as vegetables, tea, and cocoa, hence, they are known as dietary flavonoids [40].

**Figure 2:**
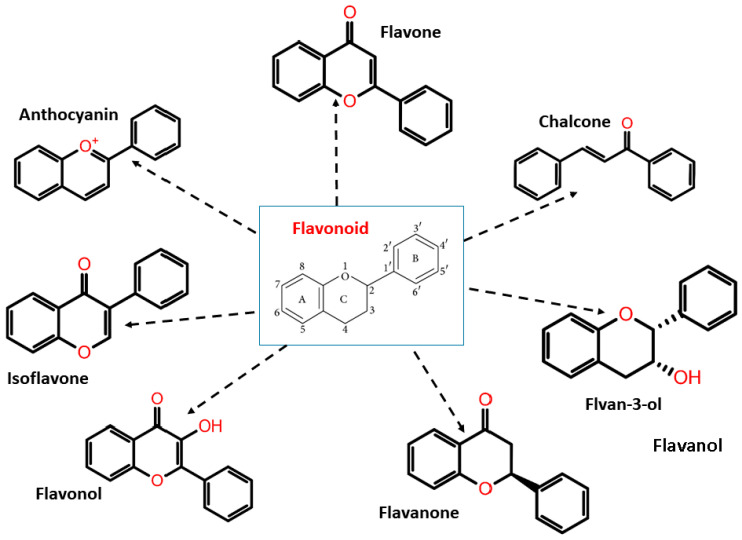
Flavonoid classification with its structure.

Flavonoids have two phenyl rings (A and B rings) linked to a heterocyclic pyrene ring (C ring having an oxygen group in it). The structure is frequently abbreviated as C6-C3-C6. Flavonoids are synthesized by the phenylpropanoid pathway, which involves frequent hydroxylation at the 5th and 7th positions on the A ring, as well as oxidation at the 3rd, 4th, or 3rd, 4th, 5th positions on the B ring [42,43,44]. Flavonoids are divided into subgroups based on the amount of carbon in the C ring and the degree of unsaturation, hydroxylation, and oxidation. Flavonoids are classified into several subclasses: flavones, flavanones, isoflavones, anthocyanins, chalcones, flavonols, flavanols/flavan-3-ols, and flavanonols (Figure 2).

### 3.2. Antimycobacterial Propert of Flavonoids

According to the reported data, flavonoids and other phenolic compounds can disrupt specific mycobacterial mechanisms that are essential for the pathogen’s survival [45] For instance, some of them impede mycolic acid synthesis, which aids in the formation of a highly impenetrable bacterial cell wall, limiting antibiotic effectiveness. Moreover, other flavonoids are reported to inhibit nucleic acid synthesis, energy metabolism, and reverse antibiotic resistance, which can improve the efficacy of currently available drugs [28] They address the issue of antitubercular drug availability and side effects, several researchers have been interested in identifying flavonoids and their derivates with anti-mycobacterial properties.

### 3.3. Flavones and Flavonols

Flavones and flavanols are a category of flavonoids commonly found in fruits, vegetables, and spices. Boonphong et al., isolated nine flavones from the root stem and bark of *Artocarpus altilis* where cycloartiocarpin, artocarpin, and chaplashin were isolated from the root stem while morusin, cudraflavone B, cycloartobiloxanthone, artonin E, cudraflavone C, and artobiloxanthone were isolated from the root bark of the plant. These isolated compounds showed antitubercular activity with a minimum inhibition concentration (MIC) ranging from 3.12 to 100 µg/mL. Artocarpin and chaplashin when compared with standard drug Kanamycin, have better MIC (3.12 µg/mL) and advocated as potent antitubercular agents [45]. Hernández et al., isolated four flavones, i.e., 5,4-dihydroxy-7-methoxyflavone, 5,8,4-trihydroxy-3,7dimethoxyflavone, 5,4-dihydroxy-3,7,8,3-tetramethoxyflavone, and 5,4-dihydroxy3,7,8-trimethoxyflavone, from the leaves of *Larrea tridentata*. The last two compounds are found to be active against multidrug-resistant TB [46]. Similarly, Murillo et al., isolated three flavones, i.e., 5-hydroxy-3,7,4-trimethoxyflavone, 5,7-dihydroxy-3,4-dimethoxyflavone, and 5,4-dihydroxy-3,7-dimethoxyflavone, from *Haplopappus sonorensis*, exhibiting anti-mycobacterial activity with MIC of 100 µg/mL against *Mtb* H37Rv strain [47]. From the roots and stems of *Derris indica*, 3-methoxy(3,4-dihydro-3,4diacetoxy)-2,2-dimethylpyrano-(7,8:5,6)-flavone, desmethoxykanugi, karanjin, pongachromene, and karanjachromene were isolated and exhibited potential activity against *Mtb* H37Rv [48]. Moreover, 7-demethylartonol E, artonin F, Linaroside, and quercetin 3,7 di-O methyl 3-sulphate are some other examples of flavones and flavanols isolated from various other plants with anti-tubercular activity [49,50,51,52]. Quercetin and rutin are the two most effective flavonols which have antimycobacterial property against *Mtb* H37Rv strain. The MICs of quercetin and rutin were found to be 6.25 µg/mL and 25 µg/mL, respectively [53]. A list of flavones and flavonols obtained from various plant are mentioned in Table 1.

### 3.4. Flavanones and Isoflavones

Chou et al. isolated flavanones, cryptoflavanones A–D, and pinocembrin, along with other compounds from the leaves of the *Cryptocarya chinensis* plant. The antituberculosis effect of these compounds was tested against *Mtb* H37Rv and *Mtb* H37Ra strains. According to the test results, pinocembrin was the most effective compound against the *Mtb* H37Rv strain with an MIC value of 3.5 µg/mL and in the case of the *Mtb* H37Ra strain, it showed antituberculosis activity with an MIC value of 12.5 µg/mL [59]. Pisonivanone, another flavanone obtained from the stem and root extract of *Pisonia aculeata*, showed antimycobacterial activity with an MIC value of 12.5 µg/mL against the *Mtb* H37Rv strain [60]. Isosakuranetin, lespedezaflavanone B, abyssinone V, butrin, isomonospermoside, etc., are some other examples of flavanone reported with antimycobacterial properties [18]. Isoflavones, such as 8,4-dimethoxy-7-O-γ,γ-dimethylallylisoflavone and maackiain isolated from *Derris indica* stems and roots, showed antimycobacterial activity against *Mtb* strains [48]. Kuete et al. reported another set of isoflavones, genisten and laburutein isolated from the stem and bark of *Ficus cordata*, for exhibiting antimycobacterial properties with MIC values of 19.53 and 4.88 µg/mL, respectively [51]. Several flavanones and isoflavones that were extracted from various plants and analyzed for their antimycobacterial properties are mentioned in the Table 2. 

### 3.5. Chalcones and Synthetic Flavonoids 

Various chalcone derivatives are designed and synthesized for studying their antitubercular activities. Pola et al. synthesized a new series of naphthyl chalcone hybrid molecules followed by synthesizing their pyrazoline derivatives by substitution of acetophenones, napthaldehyde, and hydrazine hydrate. The pyrazoline derivate compound substituted with 2-hydroxy-5-bromophenyl at the third position of pyrazoline showed potential antimycobacterial activity with MIC of 6.25 µM when compared with standard drug INH [66]. Ammaji et al. also studied the antitubercular property of chalcone by synthesizing a series of chalcone analogues. The synthesized analogues are hydroxy and chlorine-substituted chalcones [67].

The synthetic flavonoids are designed based on the substitution pattern of flavonoids. The substitution mainly includes hydroxy groups, halogens, or heteroatomic rings [68]. Compounds obtained from the flavonoid chalcones are mostly synthesized, which are significantly effective against *Mtb* [66,67,69]. Synthetic flavonoids can modulate various cellular activities such as resistance, inhibition of efflux pump, etc. For instance, Villaume et al., used synthetic flavonoids to inhibit uridine 5′-diphosphate (UDP) galactopyranose mutase (UGM) of *Mtb*. This enzyme acts as a biocatalyst during the bacterial cell wall synthesis, making potential drug target [69].

## 4. Flavonoids against Nontuberculous Mycobacteria

*Nontubercular mycobacterium* (NTM) is a pathogenic mycobacterium that does not cause tuberculosis; however, can cause pulmonary infection, skin illness, lymphadenitis, and endometritis. This group includes organisms such as *M. avium*, *M. marinum*, *M. hemophilum*, *M. gordonae*, *M. abscessus*, *M. foruitum*, and *M. chelonae.* They are typically found in conditions, such as damp soil, stream, and wetlands, and are less pathogenic than *Mtb* or *M. leprae*. The *M. avium* is the most prevalent pathogen causing pulmonary and extrapulmonary infections among the known NTM [70]. 

Flavonoids have the potential to prevent NTM growth and can be employed as a therapeutic agent for nontuberculous infections. Flavonoids suppress bacterial growth by a variety of mechanisms, including cell wall building, biofilm formation, DNA synthesis, and an efflux pump system. Flavonoids also limit bacterial growth, microbial attachment to host tissue, protein synthesis, and transport [71].

The flavanol catechin has bactericidal activity, when used against NTM. Catechin imparts oxidative damage to the cell that produces reactive oxygen species (ROS) which damages the cell membrane and also changes the cell membrane permeability [72]. Flavonoids tribuloside, afzelin, and astilbin, derived from *Heritiera littoralis* are effective against various NTM species with a minimum inhibitory concentration (MIC) of 5.0 mg/mL. These identified flavonoids inhibit the growth of NTM and are also effective as an anti-TB drug [56]. According to some studies, the growth of *M. smegmatis* can be inhibited by flavonoid 2,3,4-trihydroxy-5-methylacetophenone produced from palmyra palm, which has a MIC of 10.0 µg/mL [73]. Quercetin-3-O-β-D-glucoside obtained from *Euphoria paralias* limits the formation of biofilms and further leads to membrane disruption in *M. smegmatis* causing an outflow of intracellular components. Moreover, it has the property to inhibit glutamine synthetase as they are important enzymes involved in bacterial virulence [74]. Flavonoids including isoliquiritigenin, butein, fisetin, and 2,20,40-trihydroxychalcone inhibit *M. smegmatis* growth by targeting the dehydratase enzyme of fatty acid synthase II (FAS-II) [75]. Furthermore, another study confirmed that the D-alanine-d-alanine ligase enzyme responsible for cell wall synthesis can also be inhibited by quercetin and apigenin (4′,5,7-trihydroxyflavone) as they bind to the ATP binding pocket of the said enzyme resulting in prevention of bacterial peptidoglycan synthesis [76].

Recent studies on the anti-mycobacterial property of flavonoids showed inhibition of biofilm formation due to their structural activity and get activated when combined with antibiotics to reduce the pathogenic effect [17,77]. Biofilm formation is responsible for bacterial virulence, pathogenicity, and survival of the mycobacteria [78]. The development of biofilm in mycobacteria leads to failure of treatment, so the development should be inhibited to avoid hindrance during treatment [79]. Flavone apigenin showed antimycobacterial activities and also prevents biofilm formation [80]. Moreover, some other studies reported that C-benzylated dihydrochalcone can work as an antimycobacterial agent against *M. chelonae* and *M. fortuitum* [63]. Furthermore, platyisoflavanone, an active flavanone produced from *Platycelphium voense* is also used against *M. chelonae* with a MIC value of 23.7 mmol/L [81]. 

Various studies have proven the significance of flavonoids in the inhibition of efflux pump inhibition. Flavanone pinocembrin produced from *Alpinia katsumadai* can inhibit the efflux pump in *M. smegmatis* and also shows anti-mycobacterial activity when combined with rifampicin drug [82]. Likewise, isoflavone biochanin A also inhibits the efflux pump in *M. smegmatis* [83]. Numerous other flavonoids and their derivatives are identified that has an antimycobacterial effect on NTM. Some of them are listed in the Table 3.

## 5. Flavonoids as Potential Inhibitors of *Mycobacterium tuberculosis* Proteins In Silico Studies

In the drug discovery field, various in silico approaches such as structure-based virtual screening, pharmacophore modeling, molecular docking, molecular dynamic simulation, and absorption, distribution, metabolism, excretion, and toxicity (ADMET) analysis are utilized for identifying potent flavonoid inhibitors on various target proteins of *Mtb*. This approach has immensely helped researchers in screening and identifying flavonoids with therapeutic properties.

Swain et al. studied the antituberculosis profile of seven selected plant-based polyphenol compounds. Based on the molecular docking analysis of these seven compounds, quercetin showed the highest docking score ranging from −8 to −11 kcal/mol when compared with standard drug INH having docking scores from−5 to −7 kcal/mol, and ofloxacin having docking score from −7 to −10 kcal/mol. Quercetin shows strong molecular interaction by forming three hydrogen bonds with active site residues of the target enzyme. The drug-likeness and physicochemical analyses predicted that quercetin has a better drug profile than INH but lesser than ofloxacin [88]. Hasan et al. also confirmed the therapeutic potential of quercetin as an anti-tuberculosis drug through in silico molecular docking and ADME approach. The ADME study observed that quercetin has no carcinogenicity, mutagenicity along with low drug toxicity [89].

Using molecular docking and molecular dynamic simulation analysis, Davis et al. identified taxifolin flavanonols can be used as a dual inhibitor to inhibit *Mtb* DNA gyrase and aminoacyl-t-RNA synthetase enzyme. These two enzymes are essential for the bacterial DNA replication, transcription, and translation process. Among the eight selected flavonoid compounds, taxifolin has the best docking result with a glide score of −8.22 kcal/mol when docked with these target proteins. During molecular dynamic simulation, taxifolin remained stable at the binding site of both target proteins throughout the simulation time [90]. Flavonoids inhibiting various potential targets of *Mtb* are mentioned in Table 4.

## 6. Conclusions

According to literature analysis, more than 100 flavonoids have been identified with antimycobacterial or antitubercular properties through in vitro and in silico analyses. The emergence of drug resistance strains makes the treatment complicated and delays the expected outcome. According to reports, with the COVID-19 outbreak, TB incidence rate and notification have reduced, which further complicates the outcome of the eradication program.

In-depth studies on the various drug resistance mechanisms such as cell wall synthesis, bacterial DNA and RNA synthesis, protein synthesis, have been helpful in understanding the various druggable targets of *mycobacterium* sp., which also helped in developing new therapeutic agents. According to the literature analysis, more than 100 flavonoids isolated from various plants show antimycobacterial especially antitubercular properties. Quercetin, rutin, apigenin, catechin show significant anti-tuberculosis activity, which can be further utilized for in vivo studies. These flavonoids are effective against both tubercular and non-tubercular mycobacterium. Chalcones are utilized for the synthesis of synthetic flavonoids and showed promising results as antitubercular agents. With the growing concern of multiple drug resistance condition as well as drug toxicity, natural and synthetic flavonoids can be opted as a better alternative for treatment of TB and other mycobacterial diseases.

## Figures and Tables

**Table 1:** List of anti-tuberculosis flavones and flavonols extracted from various plants.

Plants	Family	Class	Compound	Extraction Solvent	MIC (µg/mL)	Ref.	
*Larrea tridentate*	Zygophyllaceae	Flavone	5,4′-dihydroxy3,7,8,3′- tetramethoxyflavone. 5,4′-Dihydroxy-3,7,8-trimethoxyflavone	Methanol	50	[46]
*Haplopappus* *sonorensis*	Asteraceae	Flavone	5-Hydroxy-3,7,4′-trimethoxyflavone. 5,7-Dihydroxy-3,4′-dimethoxyflavone	Methanol	100	[47]
*Derris indica*	Fabaceae	Flavone	Lacheolatin B, 3,7-Dimethoxyflavone, Pinnatin	Methanol	12	[54]
*Schinus* *terebinthifolius*	Anacardiaceae	Flavone	Apigenin	n-Hexane, methanol	23	[55]
*Lantana camara*	Verbenaceae	Flavone	Linaroside, Lantanoside	Methanol	122	[50]
*Ficus cordata*	Moraceae	Flavone	Luteolin	Methanol	100	[51]
*Ficus nervosa*	Moraceae	Flavone	Carpachromene, Apigenin	Methanol	50	[55]
*Heritiera littoralis*	Malvaceae	Flavonol	3-Cinnamoyltribuloside, Tribuloside, Afzelin	Methanol	100	[56]
*Dorstenia manni*	Moraceae	Flavonol	Dorsmanin C,D,E	Methanol	128	[57]
*Tussilago farfara*	Asteraceae	Flavonol	Quercetin	Methanol	6.25	[58]
*Arctium lappa*	Asteraceae	Flavonol	Kaempferol	Methanol	25	[58]

**Table 2:** List of anti-tuberculosis flavanones and isoflavones extracted from various plants.

Plants	Family	Class	Compound	Extraction Solvent	MIC (µg/mL)	Ref.
*Chromolaena* *odorata*	Asteraceae	Flavanone	Isosakuranetin	Methyl alcohol	25	[61]
*Pisonia* *aculeata*	Nyctaginaceae	Flavanone	Pisonivanone[(2S)-5,7,2′-Trihydroxy-8-methylflavanone]	Methanol	50	[60]
*Erythrina* *subumbrans*	Fabaceae	Flavanone	Lespedeza flavanone B, Abyssinone V, Pinocembrin	n-Hexane, Methanol	0.004, 0.06, and 2.5	[62]
*Butea* *monosperma*	Fabaceae	Flavanone	Butin, butrin, Isomonospermoside, Liquiritigenin	n-Hexane, Methanol	25, 50, 25, and 25	[52]
*Eriosema chinense Vogel*	Fabaceae	Prenylated flavanone	Khonklonginol A-H, Lupinifolinol, Dehydrolupinifolinol, Flemichin D, Eriosemaone A	n-Hexane, Dichloromethane	12.5, 1.73, and 12.5	[63]
*Ficus nervosa*	Moraceae	Flavanone	Naringenin	Methanol	2.8	[55]
*Dorstenia manni*	Moraceae	Flavanone	Dorsmanin B	Methanol	512	[57]
*Dalbergia parviflora*	Fabaceae	Isoflavone	Dalparvone	n-Hexane, Methanol	50	[64]
*Butea* *monosperma*	Fabaceae	Isoflavone	Formononetin, Afrormosin, Formononetin-7-*O*-β-d-glucopyranoside	n-Hexane, Methanol	50, 25, and 100	[52]
*Ficus nervosa*	Moraceae	Isoflavone	Prunetin, Cajanin	Methanol	30, and 110	[55]
*Rhynchosia* *precatoria*	Fabaceae	Isoflavone	Precatorin A-C, Cajanone, Lupinifolin	Methanol	≥31.25	[65]

**Table 3:** List of anti-NTM flavonoids extracted from various plants.

Plants	Family	Class	Compound	Extraction Solvent	NTM	MIC (µg/mL)	Ref.
*Euphorbia paralias*	Euphorbiaceae	Flavonoid	Quercetin-3-o-glucoside	Methanol	*M. smegmatis* and *M.* *chelonae*	3.13	[74]
*Galenia africana*	Aizoaceae	Flavone	5,7,2′-trihydroxyflavone	Ethanol	*M. abscessus*	10	[84]
*Terminalia albida*	Combretaceae	Flavonoid	Flavogallonic acid, gallagic acid	Methanol	*M. chelonae*	11	[85]
*Pelargonium reniforme*	Geraniaceae	Flavonols	Myricetin	n-Hexane, Ethyl acetate, Ethanol	*M. fortuitum*	12.5	[86]
*Lawsonia inermis*	Lythraceae	Flavonol	Lawsonicin, Kampferol, Quercetin	Methyl alcohol	*M. chelonae*	16	[85]
*Combretum apiculatum*	Combretaceae	Flavanone	Pinocembrin	Methanol	*M. fortuitum*	25	[87]
*Iris adriatica*	Iridaceae	Isoflavones	Irigenin, Irilone, Methoxylated benzophenone	Ethanol	*M. abscessus*	32	[84]
*Brassica oleracea*	Brassicaceae	Flavone	Luteolin	Methyl alcohol	*M. smegmatis*	32	[83]
*Triflolium pretense*	Fabaceae	Isoflavone	Biochanin A	n-Hexane	*M. smegmatis*	32	[83]
*Thymelea hirsuite*	Thymelaeaceae	Flavonoid	Quercetin-3-o-glucoside	Methanol	*M. smegmatis*	40	[74]

**Table 4:** List of flavonoids inhibiting potential targets.

Name of Flavonoids	Potential Targets of *Mtb*	Ref.
Naringenin and quercetin	Glutamate racemase (Murl) is responsible for the peptidoglycan synthesis.	[91]
Baicalein, pectolinarin, hispidulin, myricetin, quercetin and kaempferol.	*Mtb* proteasome required for the bacterial virulence	[92]
Quercetin	*Mtb* efflux pump	[93]
Butein, isoliquirtigenin, fisetin, 2,2′,4′-Trihydroxychalcone	Fatty acid synthase (FAS) II	[75]
Quercetin and Taxolin	DNA gyrase involved in DNA replication, transcription, and translation.	[94]
Quercetin and kaempferol	Beta-ketoacyl ACP synthase III, involved in mycolic acid synthesis	[95]
Quercetin-3-O-β-d-glucoside	*M. tuberculosis* glutamine synthetase (MtGS) responsible for the pathogenesis.	[74]
Luteolin and Quercetin	Uridine 5′-diphosphategalactopyranosemutase (UGM) involved in cell wall biosynthesis	[69]
Taxolin	Aminoacyl-t-RNA synthetase involved in DNA replication, transcription and translation	[90]
